# Supplementary material for: LncRNA JPX Promotes Esophageal Squamous Cell Carcinoma Progression by Targeting miR-516b-5p/VEGFA Axis
Source: Cancers (Basel). 2022 May 31;14(11):2713. doi: 10.3390/cancers14112713 (PMC9179376; doi:10.3390/cancers14112713)
Supplement: Supplementary file 1 [file cancers-14-02713-s001.zip › Supplementary Figures and legends.pdf]

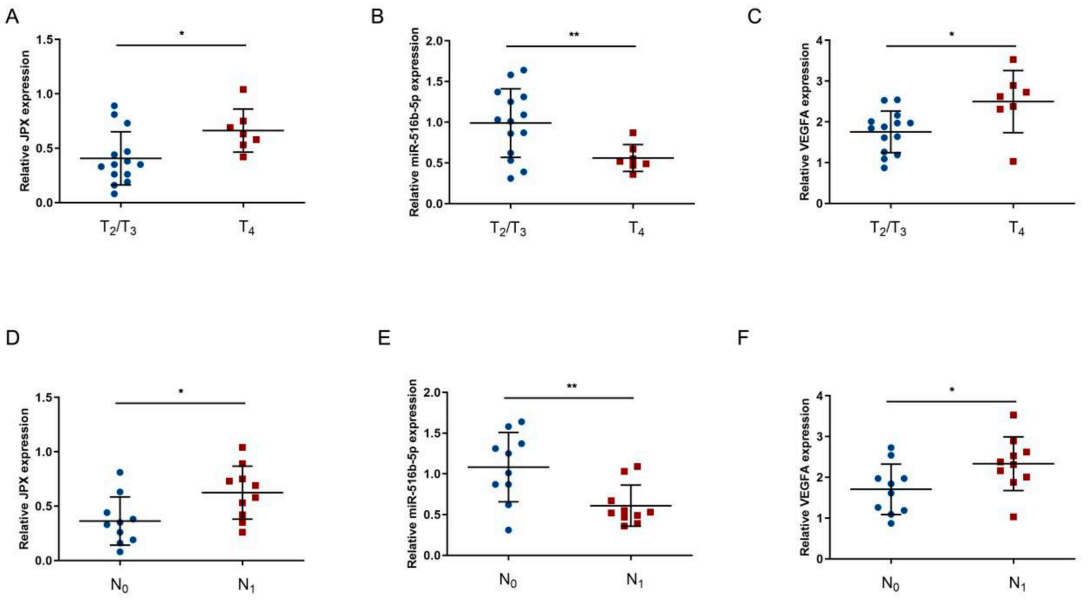

Supplementary Figure S1. Clinical analysis of JPX, miR-516b-5p, and VEGFA for ESCC patients. Expression of JPX (A), miR-516b-5p (B), and VEGFA (C) in patients with different T stages of ESCC. Expression of JPX (D), miR-516b-5p (E), and VEGFA (F) in patients with different N stages of ESCC. **P*< 0.05, ***P*< 0.01. The statistical differences were calculated by Student's t-test.

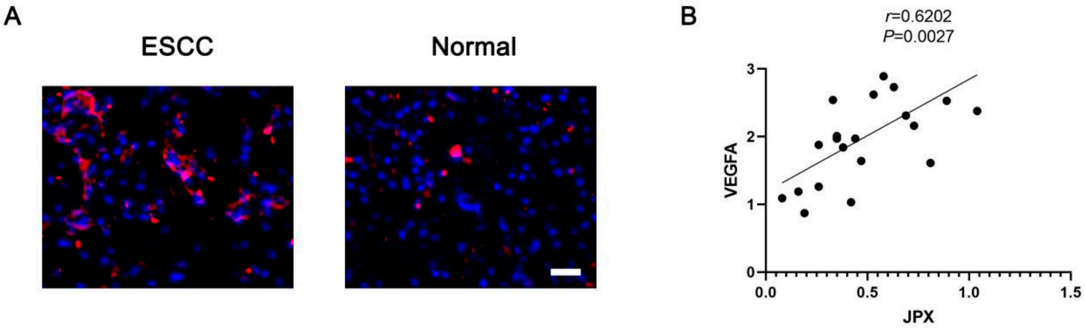

Supplementary Figure S2. LncRNA JPX expression in ESCC tissues. The expression of JPX (red) in ESCC tissues or normal tissues was determined by FISH assay. Nuclei was stained by DAPI (blue). Scale bar, 50 μm. (B) The coefficient between JPX and VEGFA in ESCC patients.
