# Supplementary material for: LncRNA JPX Promotes Esophageal Squamous Cell Carcinoma Progression by Targeting miR-516b-5p/VEGFA Axis
Source: Cancers (Basel). 2022 May 31;14(11):2713. doi: 10.3390/cancers14112713 (PMC9179376; doi:10.3390/cancers14112713)
Supplement: Supplementary file 1 [file cancers-14-02713-s001.zip › Table S1.pdf]

Supplementary Table S1. Correlation between JPX expression and clinicopathological characteristics of ESCC patients.

Clinicopathological parameter		JPX expression		P-Value[a]
		High expression	Low expression	
Age (year)	<60	10	3	0.6972
	⩾60	6	2	
Gender	Female	3	2	0.4626
	Male	13	3	
Tumor location	Upper	2	1	0.8886
	Middle	6	2	
	Lower	8	2	
Tumor grade*	Well	0	2	0.0282
	Moderate	4	1	
	Poor	12	2	
TNM stage*	I	0	2	0.0159
	II	6	0	
	III	10	3	

[a] Chi-square test results, * $P<0.05$.
