# Supplementary material for: LncRNA JPX Promotes Esophageal Squamous Cell Carcinoma Progression by Targeting miR-516b-5p/VEGFA Axis
Source: Cancers (Basel). 2022 May 31;14(11):2713. doi: 10.3390/cancers14112713 (PMC9179376; doi:10.3390/cancers14112713)
Supplement: Supplementary file 1 [file cancers-14-02713-s001.zip › Table S2.pdf]

Supplementary Table S2. Primers used for qRT-PCR, siRNA, mimics sequences.

Name	Sequence (5'-3')
**qRT-PCR**	
JPX-F	TTGCAAGGCGTCCGAAGTAT
JPX-R	TTGTACCACCGTCATCAGGC
miR-516b-5p-F	CAGATCTGGAGGTAAGAAGCACTTT
miR-516b-5p-R	AAAGTGCTTCTTACCTCCAGATCTG
VEGFA-F	GGCAAAAACGAAAGCGCAAG
VEGFA-R	ATTAGACAGCAGCGGGCAC
GAPDH-F	GAGAAGGCTGGGGCTCATTT
GAPDH-R	GAGAAGGCTGGGGCTCATTT
U6-F	GCAGACCGTTCGTCAACCTA
U6-R	AATTCTGTTTGCGGTGCGTC
**siRNAs**	
si-NC (JPX)	GGAGAATAATTTCTTTCTUU
si-JPX#1	GUGCUGAGCAGUUGUCAUAUU
si-JPX#2	GCGAGACUCUGUCUCUAAAUU
si-NC (VEGFA)	GCGAGAAGTGCTAGCTCGUU
si-VEGFA#1	GGCAGCUUGAGUUAAACGAUU
si-VEGFA#2	CGAGGCAGCUUGAGUUAAAUU
**Mimics/control**	
miR-516b-5p mimics (sense)	AUCUGGAGGUAAGAAGCACUUU
miR-516b-5p mimics (antisense)	AGUGCUUCUUACCUCCAGAUUU
miR-516b-5p mimics control (sense)	UGCUUGCAACGUUUCACGATT
miR-516b-5p mimics control (antisense)	UCGUGAAACGUUGCAAGCATT
