# Supplementary figures and images for: LncRNA JPX Promotes Esophageal Squamous Cell Carcinoma Progression by Targeting miR-516b-5p/VEGFA Axis
Source: Cancers (Basel). 2022 May 31;14(11):2713. doi: 10.3390/cancers14112713 (PMC9179376; doi:10.3390/cancers14112713)

Figure S3 Original Western Blot

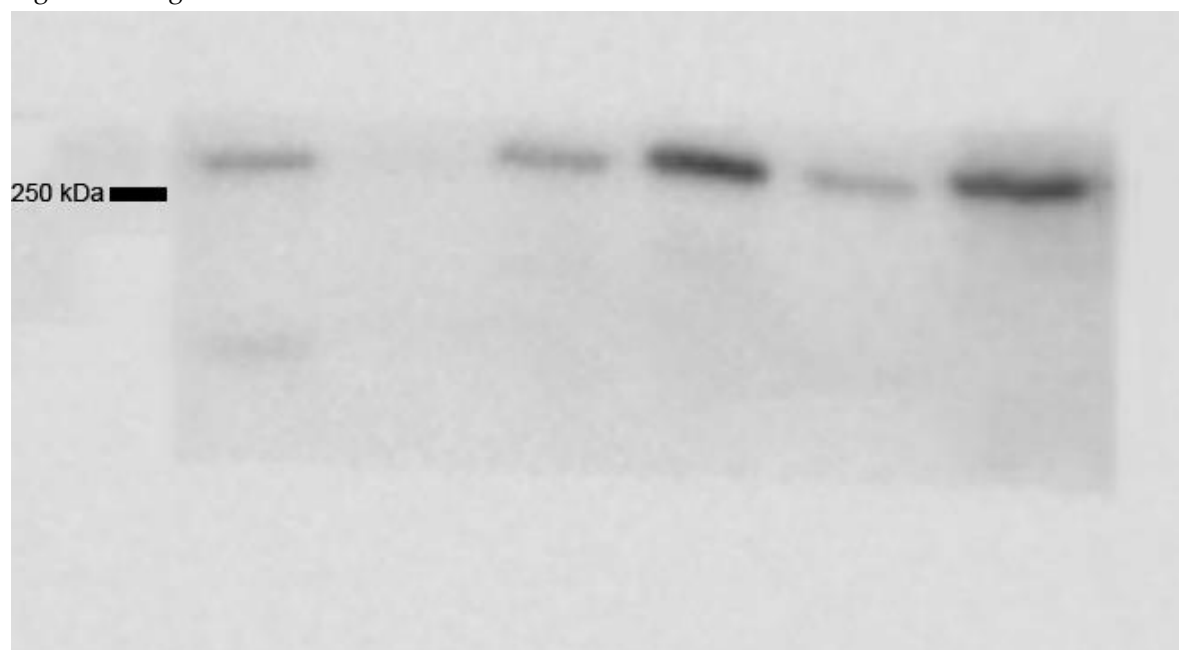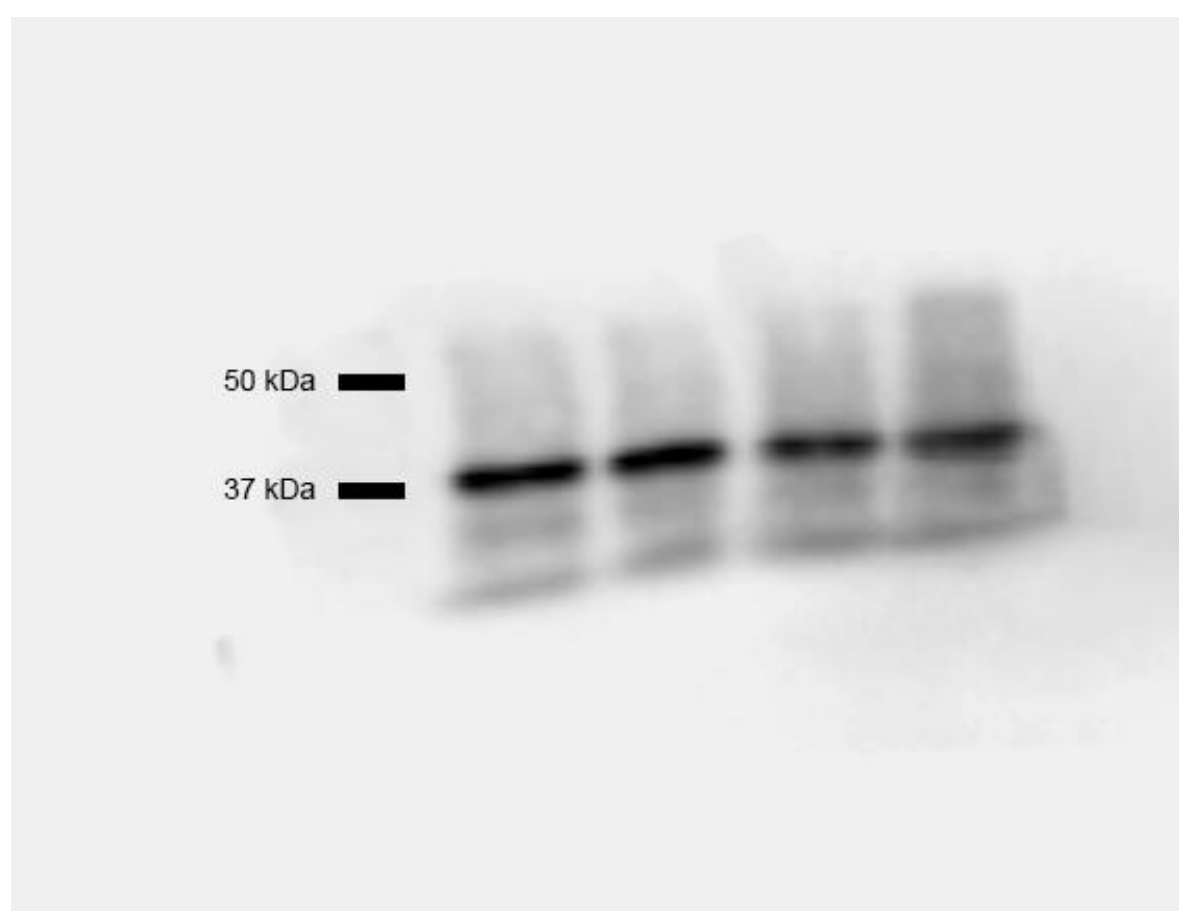

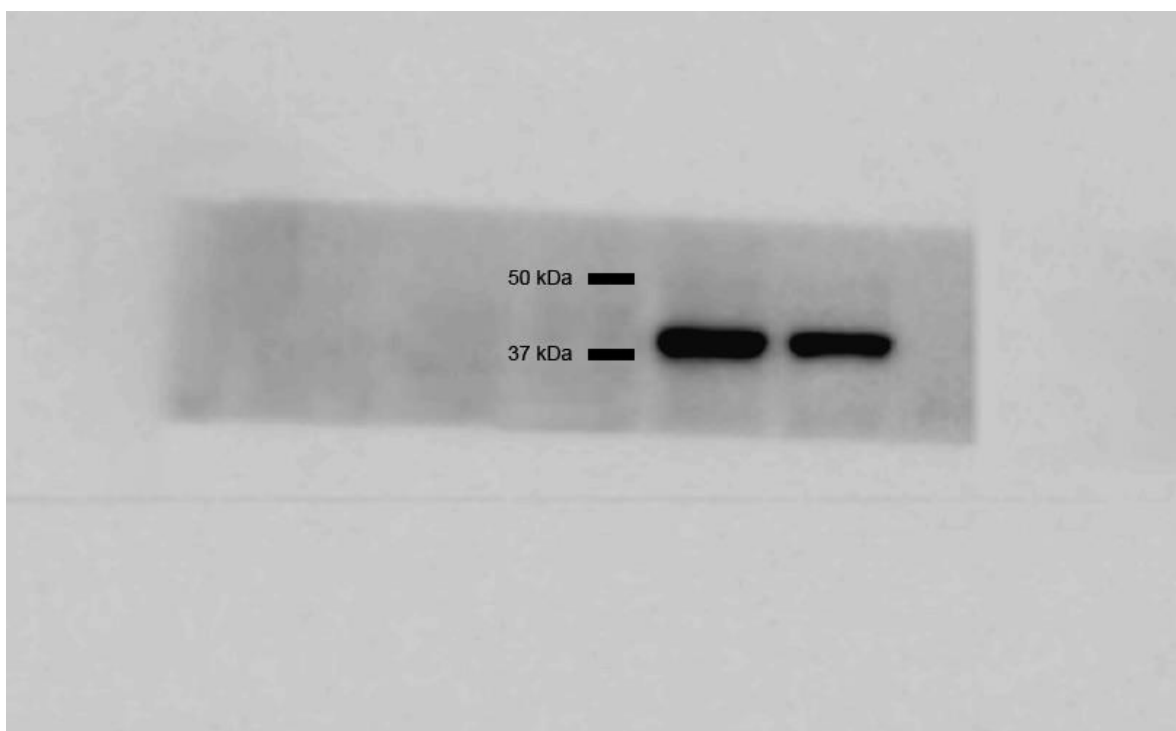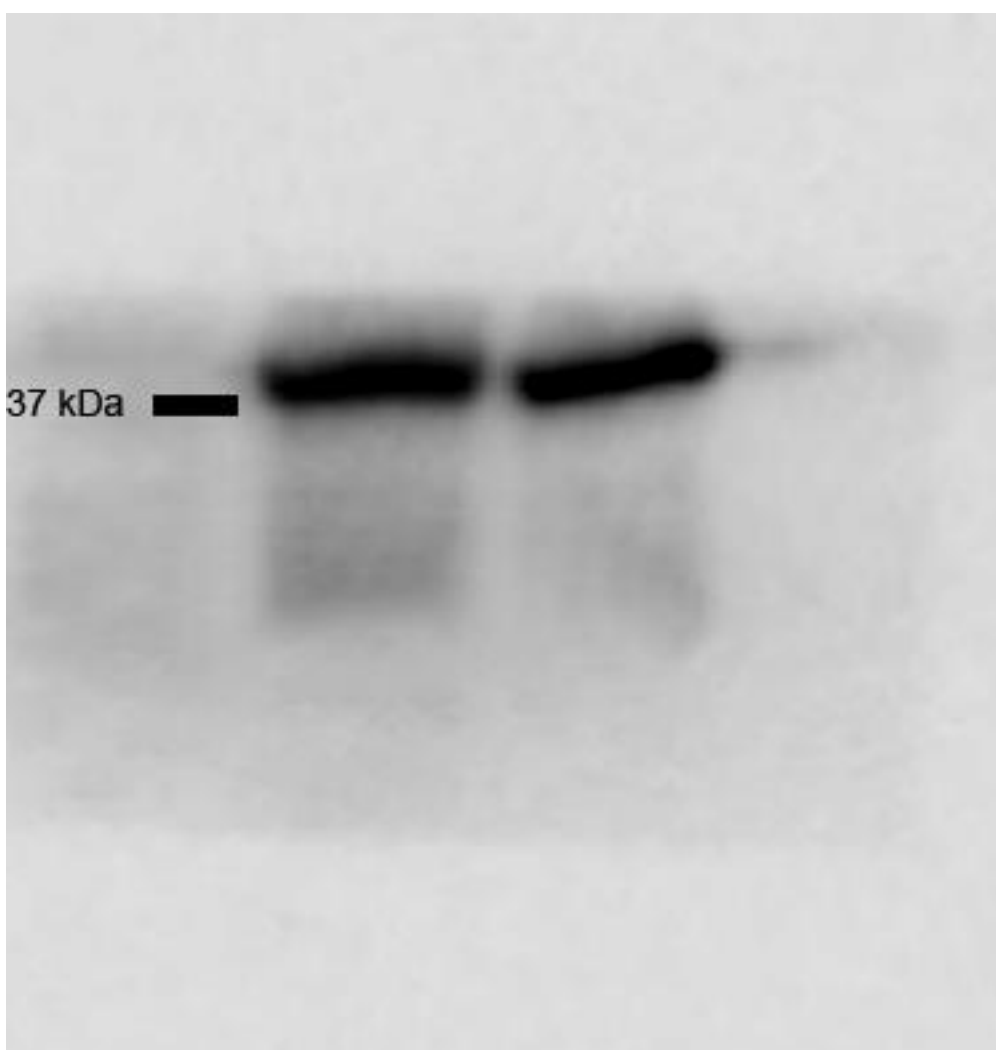

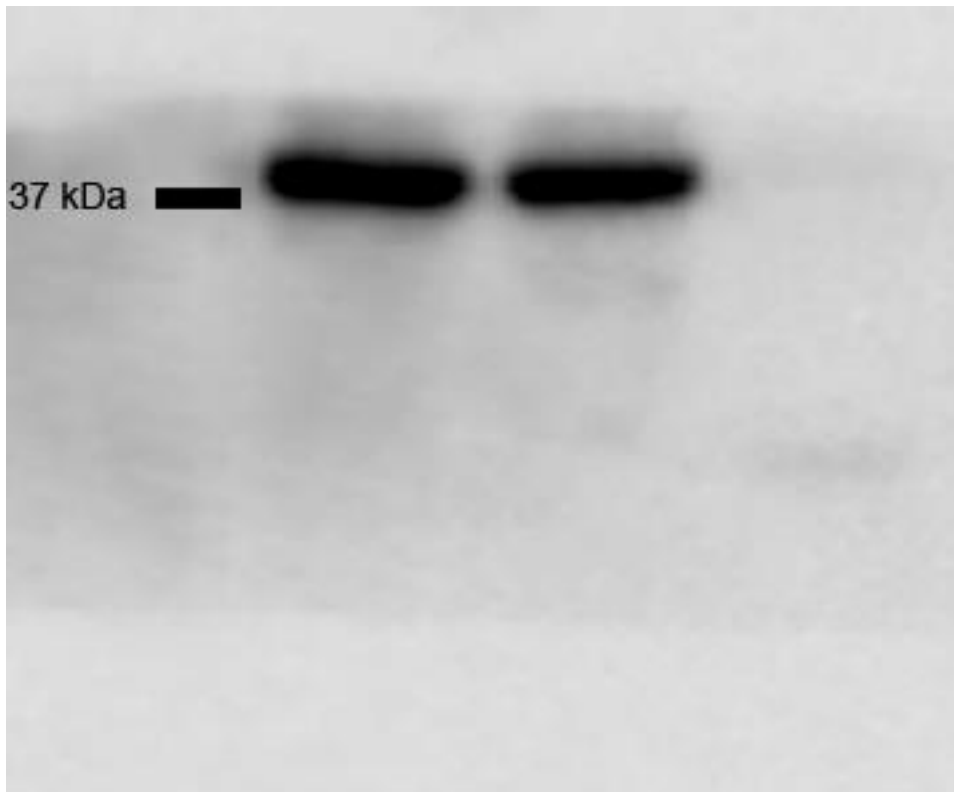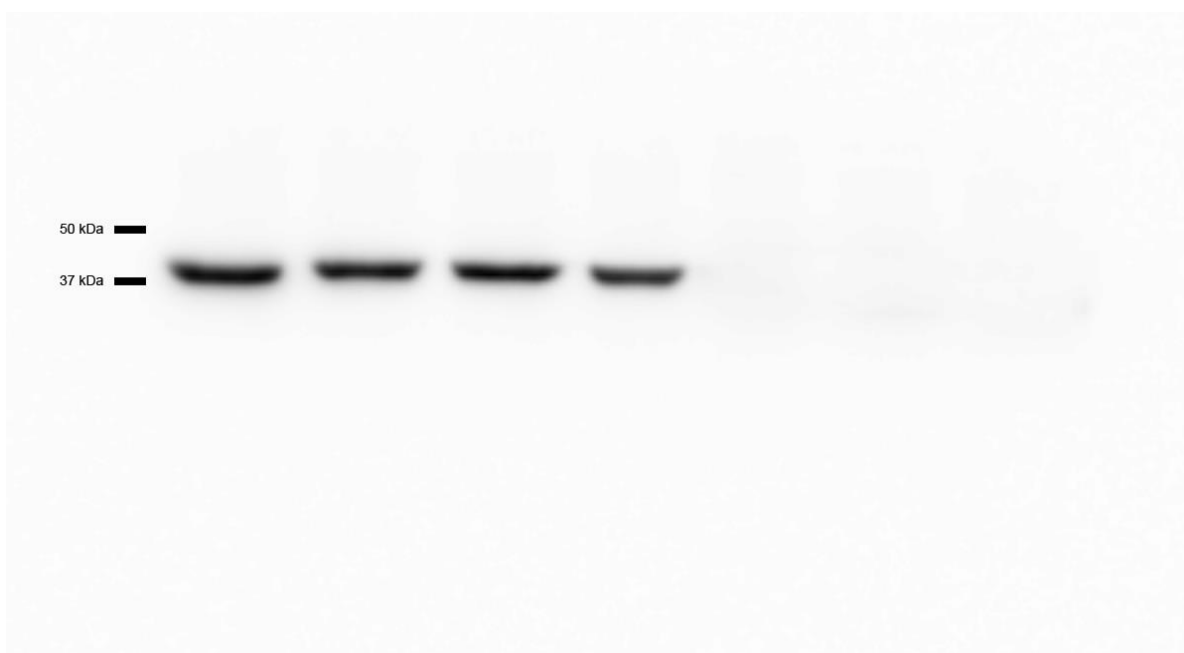

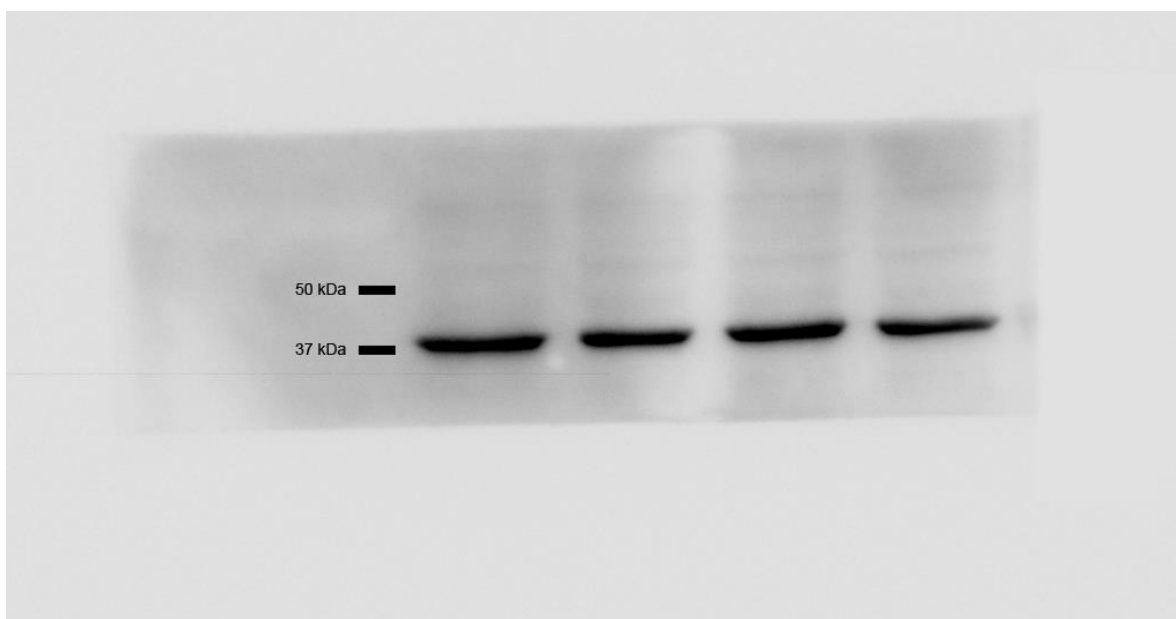

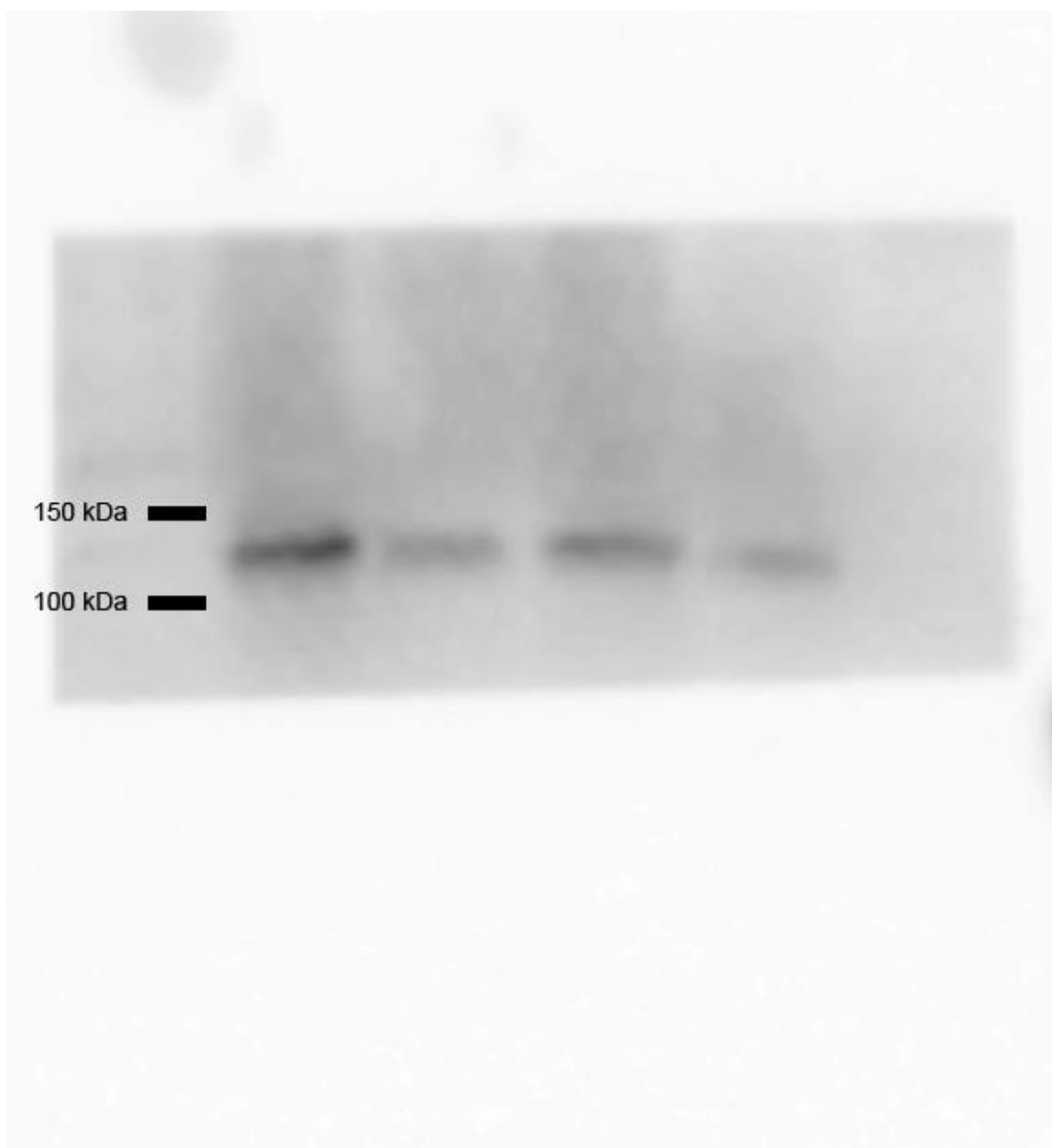

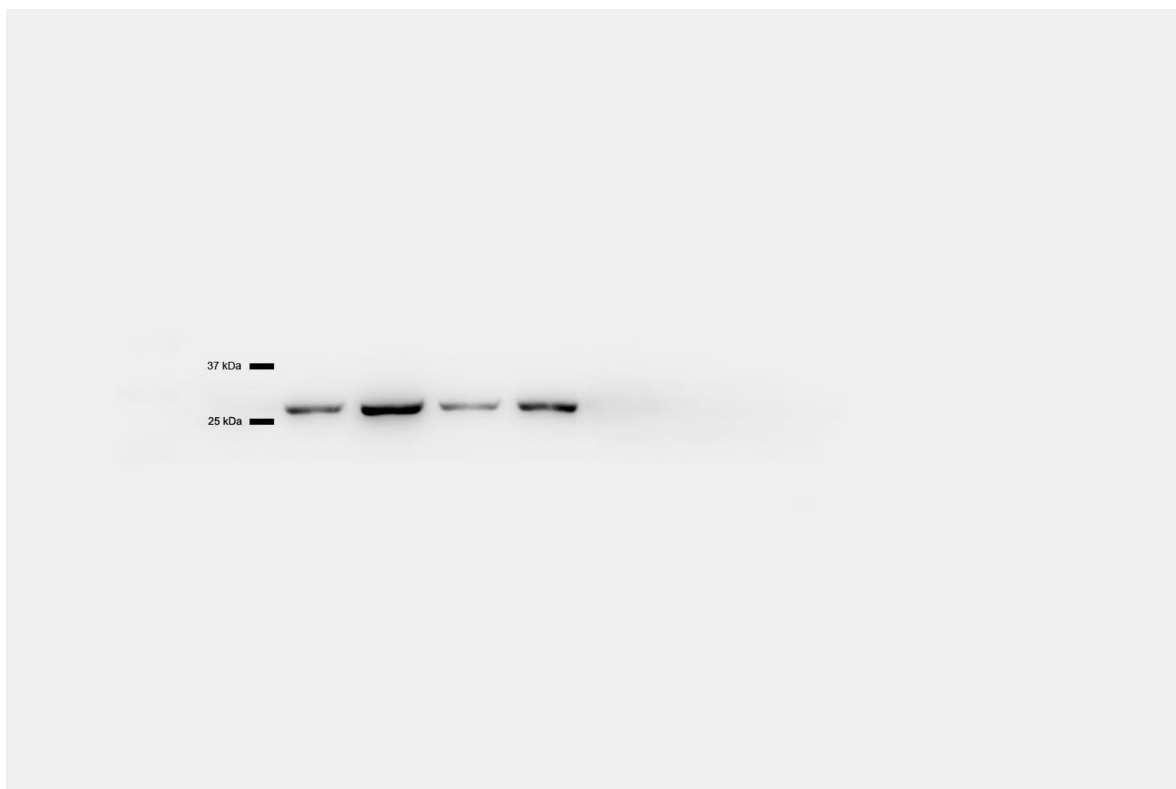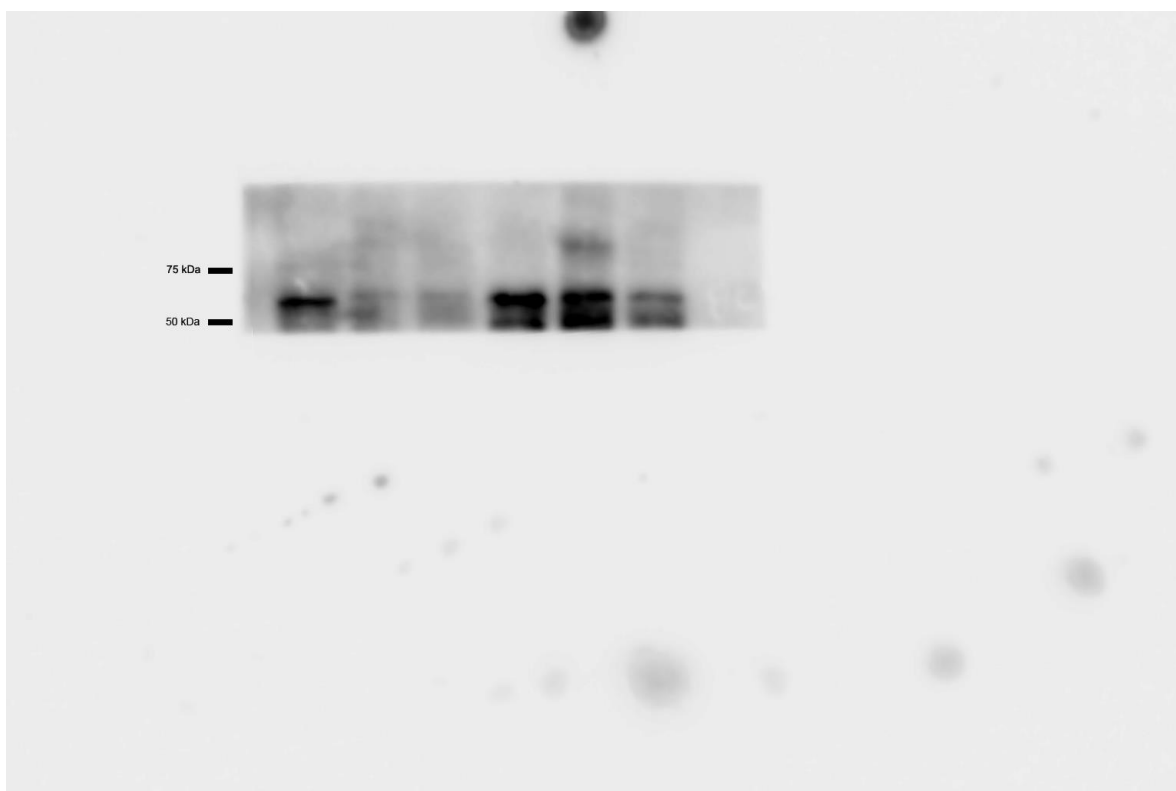

Supplement: Supplementary file 1 [file cancers-14-02713-s001.zip › FigureS3 Original Western Blot.pdf]
